# Both viable *Bifidobacterium longum* subsp. *infantis* B8762 and heat-killed cells alleviate the intestinal inflammation of DSS-induced IBD rats

**DOI:** 10.1128/spectrum.03509-23

**Published:** 2024-04-22

**Authors:** Zhaojie Li, Chuantao Peng, Yaru Sun, Tao Zhang, Cuijiao Feng, Weiqin Zhang, Tian Huang, Guoqiang Yao, Heping Zhang, Qiuwen He

**Affiliations:** 1 Key Laboratory of Dairy Biotechnology and Engineering, Ministry of Education, Inner Mongolia Agricultural University, Hohhot, China; 2 College of Food Science and Engineering, Qingdao Agricultural University, Qingdao, China; 3 Qingdao Special Food Research Institute, Qingdao, China; 4 Key Laboratory of Dairy Products Processing, Ministry of Agriculture and Rural Affairs, Inner Mongolia Agricultural University, Hohhot, China; 5 Inner Mongolia Key Laboratory of Dairy Biotechnology and Engineering, Inner Mongolia Agricultural University, Hohhot, China; Universita degli Studi di Parma, Parma, Italy

**Keywords:** inflammatory bowel disease, *Bifidobacterium longum *subsp.* infantis *B8762, postbiotics, gut microbiota, immunity, fecal metabolome

## Abstract

In view of the safety concerns of probiotics, more and more attention is paid to the beneficial effects of dead probiotics cells. Herein, we investigated and compared the alleviation effects of viable *Bifidobacterium longum* subsp. *infantis* B8762 (*B. infantis* B8762) and its heat-killed cells on dextran sodium sulfate (DSS)-induced inflammatory bowel disease (IBD) rats. Four groups of rats (*n* = 12 per group) were included: normal control, DSS-induced colitis rats without bacterial administration (DSS), DSS-induced colitis rats with viable *B. infantis* B8762 administration (VB8762), and DSS-induced colitis rats with dead *B. infantis* B8762 administration (DB8762). Our results showed that both VB8762 and DB8762 administration exerted significant protective effects on DSS-induced IBD rats, as evidenced by a reduction in mortality, disease activity index score, body weight loss, as well as decreased histology score, which were companied by a significant decrease in serum pro-inflammatory factors compared with DSS group, and a stronger effect on modulating the fecal microbiota alpha-diversity and beta-diversity compared with DSS group. Additionally, the fecal metabolome results showed that both VB8762 and DB8762 interventions indeed altered the fecal metabolome profile and related metabolic pathways of DSS-induced IBD rats. Therefore, given the alleviation effects on colitis, the DB8762 can be confirmed to be a postbiotic. Overall, our findings suggested that VB8762 and DB8762 had similar ability to alleviate IBD although with some differences. Due to the minimal safety concern of postbiotics, we propose that the postbiotic DB8762 could be a promising alternative to probiotics to be applied in the prevention and treatment of IBDs.

**IMPORTANCE:**

Inflammatory bowel disease (IBD) has emerged as a global disease because of the worldwide spread of western diets and lifestyles during industrialization. Up to now, many probiotic strains are used as a modulator of gut microbiota or an enhancer of gut barrier to alleviate or cure IBD. However, there are still many issues of using probiotics, which were needed to be concerned about, for instance, safety issues in certain groups like neonates and vulnerable populations, and the functional differences between viable and dead microorganisms. Therefore, it is of interest to investigate the beneficial effects of dead probiotics cells. The present study proved that both viable *Bifidobacterium longum* subsp. *infantis* B8762 and heat-killed cells could alleviate dextran sodium sulfate-induced colitis in rats. The findings help to support that some heat-killed probiotics cells can also exert relevant biological functions and can be used as a postbiotic.

## INTRODUCTION

The Food and Agriculture Organization of the United Nations-World Health Organizatio, define the term “probiotic” as “live microorganisms which, when administered in adequate amounts, confer a health benefit on the host” (1). Many studies have powerfully verified that probiotics possess many beneficial functions such as cholesterol-lowering, anti-cancer, anti-hypertension effects, neurodegenerative and viral diseases prevention, and relief of diarrhea, constipation, inflammatory bowel disease (IBD), and irritable bowel syndrome (IBS) (2, 3), etc. Of those, the improvement or even cure of gastrointestinal diseases including acute infectious diarrhea, ulcerative colitis (UC), IBD, IBS, antibiotic-associated diarrhea, functional gastrointestinal disorders and necrotizing enterocolitis is noteworthy (4, 5). It makes senses that the use of probiotics can directly influence gut microbiota to obtain clinical benefits. Gut microbiota, containing more than 1,000 different microbial species, embracing 100 trillion microorganisms, interface with mucosal surfaces directly and are key contributors to immune regulation, energy metabolism, and epithelial barrier maintenance (6, 7). It has been reported that probiotics can regulate the inflammatory response by stimulating cytokine production (8, 9). Particularly, some strains of *Lactobacillus* and *Bifidobacterium* genera can promote host immunity by activating macrophages, natural killer cells, and T lymphocytes (10–12). Furthermore, among the main effects of probiotics at the intestinal level, the following are outstanding: protection against pathogens, balance and restoration of the gut microbiota, and enhancement of the intestinal epithelial cell survival by maintaining intestinal barrier integrity and immunomodulation (13–15).

As a chronic recurrent intestinal non-specific inflammatory disease, the incidence of IBD is increasing globally year by year, and its common clinical types include UC, Crohn’s disease (CD), and IBD unclassified (16). An evaluation of European countries studies revealed that the current mean prevalence of IBD in the total population is estimated at 1/1,000 (17). The overall incidence of IBD in China is 1.74 per 100,000 persons per year, among which the incidence of IBD in children has been increasing year by year (18). According to the World Gastroenterology Organization, IBD has emerged as a global disease because of the worldwide spread of western diets and lifestyles during industrialization (19, 20). Although the precise etiology and pathogenesis of IBD remain to be fully demonstrated, it is likely to involve many factors including unknown environmental factor, genetic susceptibility, intestinal flora imbalance, impaired intestinal barrier function, and immune response disorders (21–24). Of late, the potential role of the gut microbiota in the development, progression, and treatment of IBD has been a subject of considerable interest and inquiry. More and more studies in animal models or human subjects have shown that the gut microbiome is different in IBD patients compared with that in healthy control subjects (25). Combined with other evidence, suffice it to say that there is substantial, if not overwhelming, evidence for a fundamental role of the gut microbiota and its interactions with the host immune system in IBD (26–28). Therefore, exploring an effective gut microbiota modulator may provide new therapies in IBD.

Up to now, many probiotic strains are used as a modulator of gut microbiota or an enhancer of gut barrier, and so have been proven to alleviate or cure IBD in animal models or patients (4, 29–31). However, there are still many issues needed to be addressed, for instance, safety issues with the use of live strains in certain groups, such as neonates (32, 33) and vulnerable populations (34), or the lack of studies evaluating the viability of microorganisms once in the intestine and the functional differences between viable and dead microorganisms (35), which result in an increased interest to use heat-killed probiotics (36, 37). Much published evidence has proved that heat-treated culture preparations containing dead cells and their metabolites can also exert relevant biological functions, such as restoring the normal intestinal homeostasis, which are similar to those with live cells, although with potential differences (36, 37). After inactivation mainly by heat treatment, dead cells can release bacterial components with key immunomodulating effects and with antibacterial activities against pathogens. Different bacterial components, such as peptidoglycans, lipoteichoic acids, and exopolysaccharides, have been proven to be mainly involved in these properties (37, 38). Beneficial properties of heat-killed bacteria elucidated their benefits in different indications, such as in neonates, without incurring the risks associated with live microorganisms, and with advantages in terms of transport and storage.

*Bifidobacterium longum* subsp. *infantis* B8762 (*B. infantis* B8762) is isolated from infant feces in Hohhot City of Inner Mongolia Autonomous Region, China. In the present study, we aimed to investigate if *B. infantis* B8762 or heat-killed cells can alter gut microbiota, affect metabolites profile of feces, enhance immune responses, as well as improve intestinal inflammation in IBD rat models. Meanwhile, we are also interested in the differences between viable *B. infantis* B8762 and heat-killed cells in the alleviation of the intestinal inflammation of IBD rats.

## RESULTS

### Both viable and dead *B. infantis* B8762 alleviated the symptoms of dextran sodium sulfate (DSS)-induced colitis

**Fig 1:**
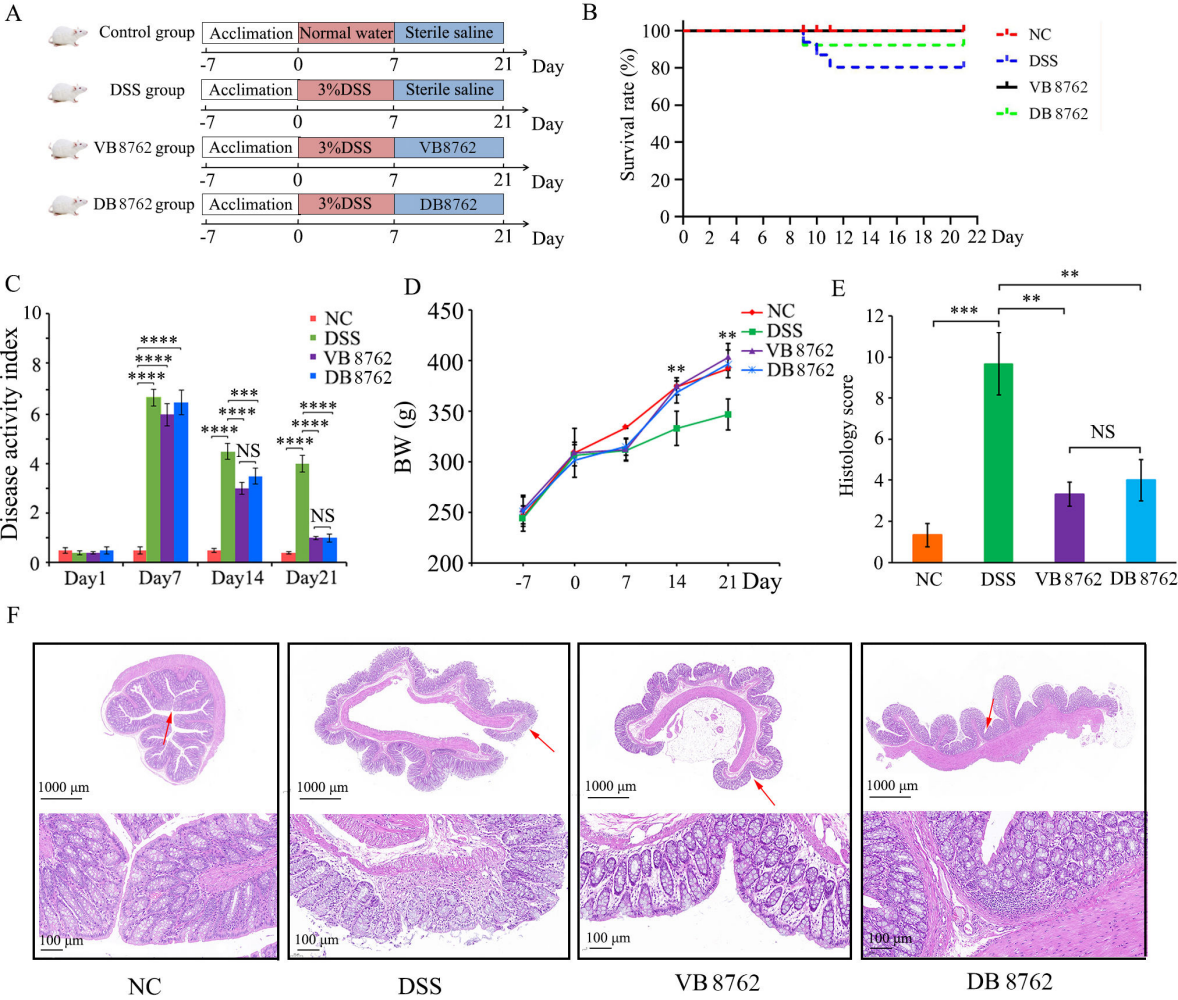
Both viable and dead *B. infantis* B8762 administration alleviated DSS-induced colitis. (**A**) Scheme of the experimental design. (**B**) Survival rate. (**C**) Disease activity index and (**D**) body weight change during the experiment period. (**E**) Histology score. (**F**) Hematoxylin and eosin staining micrographs of colon tissues; microscopic images (100× magnification) are a magnified image of the area indicated by the red row. Error bars represent standard deviation of the mean. *P < 0.05; **P < 0.01; ***P < 0.001; ****P < 0.0001.

To evaluate the beneficial effects of viable and dead *B. infantis* B8762, a DSS-induced colitis rat model was built (Fig. 1A). In this animal trial, no rat died both in the normal control (NC) and VB8762 groups, and one rat in the DB8762 group died on day 8 with a final survival rate of 91.7%, but three rats in the DSS group died on the 8th, 9th, 10th day, respectively, with a final survival rate of 75% (Fig. 1B). This result indicated that VB8762 and DB8762 could decrease the mortality caused by DSS. As shown in Fig. 1C, a 7-day DSS inducement resulted in significantly higher disease activity index (DAI) scores than NC group, but no significant difference was observed among the DSS, VB8762, and DB8762 groups, indicating the success of DSS-induced colitis rat model. During the period of microbial administration (8th to the 21st day), the DAI scores of DSS, VB8762, and DB8762 groups all showed a downward trend. But compared with the DSS group, VB8762 or DB8762 administration significantly reduced the DAI scores (on day 14, *P* < 0.0001 and *P* < 0.001, respectively; on day 21, *P* < 0.0001 and *P* < 0.0001, respectively). The NC group displayed normal weight gains during the DSS-induced period (0–7 days), but there were almost no weight gains in the DSS-challenged rats of the DSS, VB8762, and DB8762 groups. During the microbial administration period, there were significant body weight increases in both VB8762 and DB8762 groups compared with DSS group (*P* < 0.01, Fig. 1D), suggesting that both VB8762 and DB8762 administrations could obviously improve DSS-induced body weight loss.

The DSS group showed higher colon histopathological score than the NC (*P* < 0.001), VB8762 (*P* < 0.01), and DB8762 groups (*P* < 0.01) (Fig. 1E). The histopathological changes in rat colon tissues stained with hematoxylin and eosin (H&E) were shown in Fig. 1F. The colon crypt structure of rats in the NC group was regular, with neatly arranged glands, abundant cup cells, intact mucosal, submucosal, muscle, and plasma membrane layers, and without obvious inflammatory cell infiltration. However, the local colon mucosal layers of rats in the DSS group were detached or damaged severely, the crypt structure was atrophied and unremarkable, and the cup cells decreased. In contrast, in rats administered with VB8762 or DB8762, the colon crypt structures were entire and showed similar histological features to those of rats in the NC group, with only a small amount of inflammatory cell infiltration in the DB8762 group.

### Both viable and dead *B. infantis* B8762 downregulated pro-inflammatory cytokines in DSS-induced IBD rats

**Fig 2:**
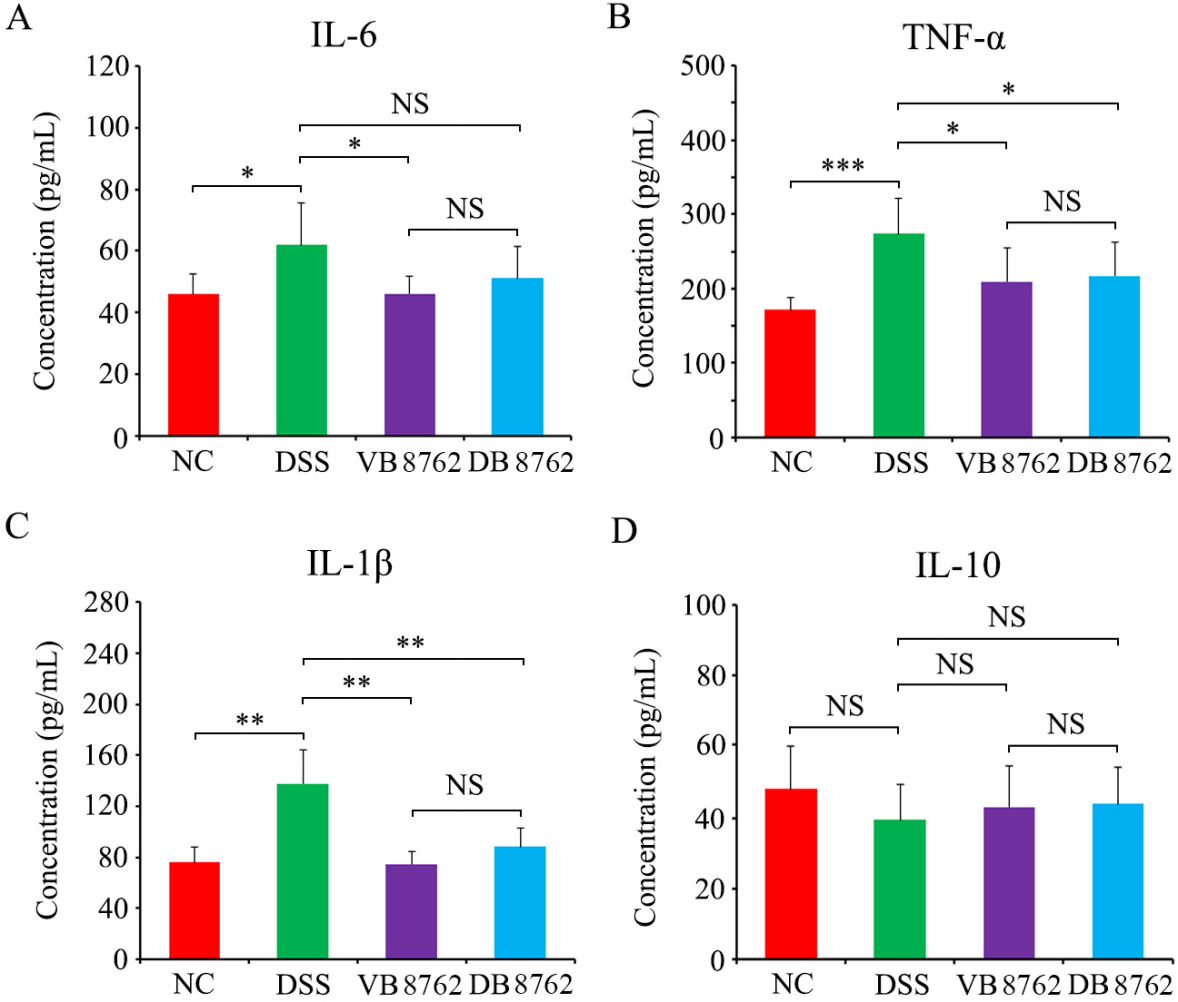
Differences in serum cytokine levels in the NC, DSS, VB8762, and DB8762 groups. Levels of IL-6 (**A**), TNF-α (**B**), IL-1β (**C**), and (**D**) IL-10 in four experimental groups. Error bars represent standard deviation of the mean. *P < 0.05; **P < 0.01; ***P < 0.001.

Three pro-inflammatory cytokines interleukin-6 (IL-6), tumor necrosis factor-α (TNF-α), and IL-1β and one anti-inflammatory cytokine IL-10 in the serum in each group were measured on day 21. As shown in Fig. 2A through C, the levels of all three pro-inflammatory cytokines of the DSS group significantly increased (IL-6, *P* < 0.05; TNF-α, *P* < 0.001; IL-1β, *P* < 0.01) compared with those of the NC group. But all these cytokines of the VB8762 group were significantly lower (IL-6, *P* < 0.05; TNF-α, *P* < 0.05; IL-1β, *P* < 0.01) than those of the DSS group. In the DB8762 group, except IL-6 (*P* > 0.05), the two cytokines of TNF-α and IL-1β were both significantly lower (TNF-α, *P* < 0.05; IL-1β, *P* < 0.01) than those of the DSS group. Surprisingly, there were no significant differences (*P* > 0.05) in the IL-10 levels between the DSS and the NC, VB8762, or DB8762 groups, indicating VB8762 or DB8762 administration cannot significantly increase the IL-10 level. These results further proved that both VB8762 and DB8762 administration could alleviate the inflammation symptoms in IBD rats by downregulating the pro-inflammatory cytokines

### Both viable and dead *B. infantis* B8762 restored the gut microbiota compositional structure disrupted by DSS

**Fig 3:**
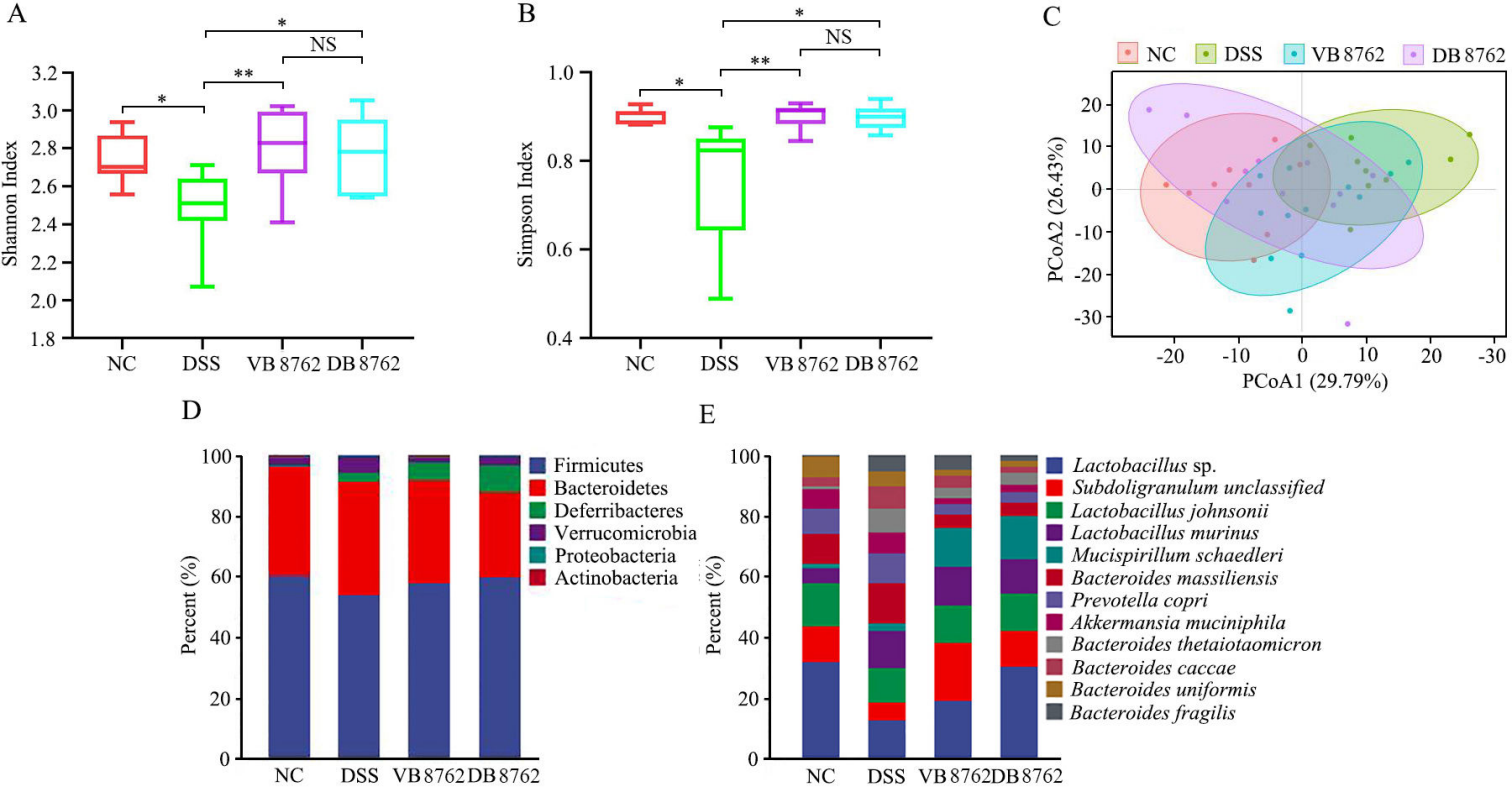
The taxonomic gut microbiota of NC, DSS, VB8762, and DB8762 groups. (**A**) Shannon index. (**B**) Simpson index. (**C**) PCoA (Bray-Curtis distance) score plot of rat gut microbiota. (**D**) Phylum-level gut microbiota profile. (**E**) Species-level (top 12 species) gut microbiota profile. Error bars represent standard deviation of the mean. *P < 0.05; **P < 0.01.

To determine whether VB8762 or DB8762 administration altered the gut microbiota, whole-metagenome shotgun sequencing analysis was performed on fecal samples of the NC, DSS, VB8762, and DB8762 groups collected at the end of the trial. We originally measured gut microbial alpha-diversity by Shannon index and Simpson index. Consistently, the two indices manifested similar tendencies and found that the fecal microbiota of the DSS group had significantly lower values of Shannon and Simpson indices compared with those of the NC group, and both VB8762 and DB8762 administration could mitigate the DSS-induced reduction in gut microbial diversity (*P* < 0.01 and *P* < 0.05 for Shannon index, respectively; *P* < 0.01 and *P* < 0.05 for Simpson index, respectively; Fig. 3A and B). To further understand the role of microbiome diversity, we analyzed beta-diversity by principal coordinates analysis (PCoA; Bray-Curtis distance). Symbols representing NC and DSS-treated rats with or without strain treatment showed distinct clustering pattern on the PCoA plot (Adonis test, NC versus DSS, *R*^2^ = 0.3878, *P* = 0.001; NC versus VB8762, *R*^2^ = 0.2364, *P* = 0.001; NC versus DB8762, *R*^2^ = 0.2320, *P* = 0.001; Fig. 3C), suggesting apparent differences in the fecal microbiota compositional structure between control and DSS-treated groups. Similar analyses were performed between the DSS and VB8762 groups, as well as the DSS and DB 8762 groups. Significant difference in the fecal microbiota structure was observed in both analysis cases (VB8762 versus DSS, *R*^2^ = 0.1808, *P* = 0.001; DB8762 versus DSS, *R*^2^ = 0.1828, *P* = 0.002), suggesting that both VB8762 and DB8762 administration could restore the gut microbiota diversity which was disrupted by DSS treatment. Although there was a large proportion of overlap between the VB8762 group and DB8762 groups, significant difference still existed between the two groups (*R*^2^ = 0.0886, *P* = 0.042), suggesting VB8762 has a little higher ability than DB8762 in restoring the gut microbiota diversity.

**Fig 4:**
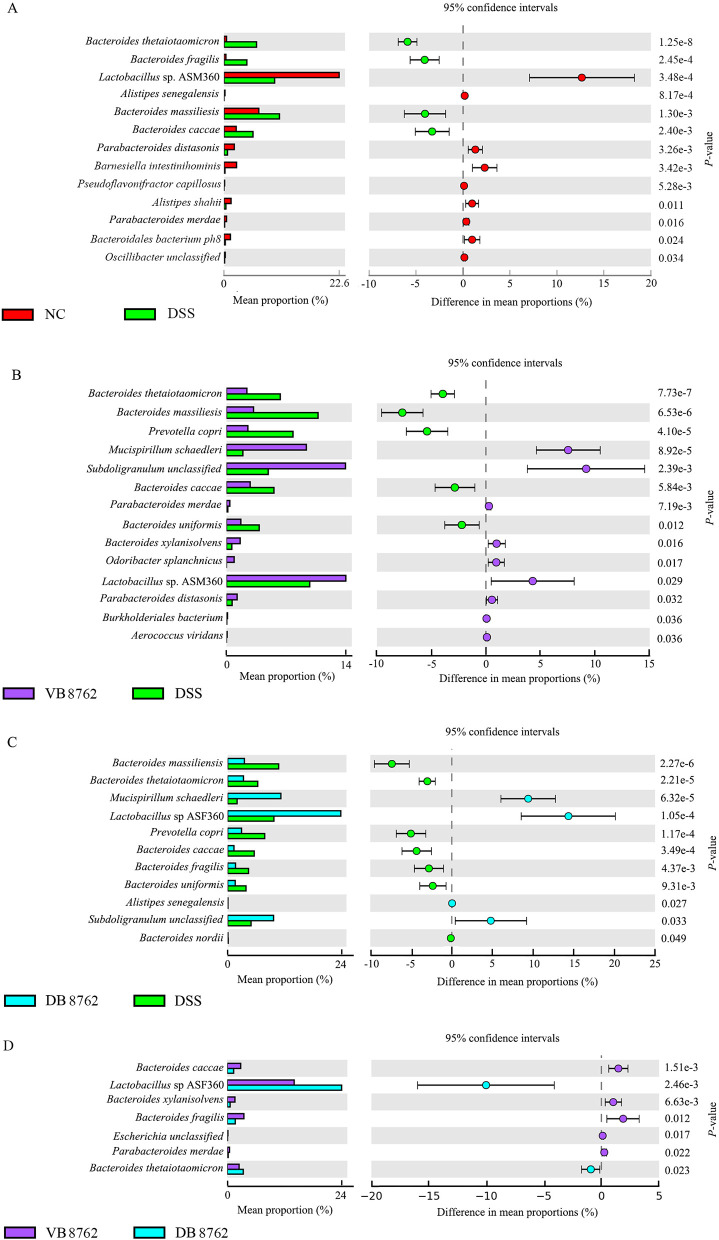
Differential species of four pairwise comparisons. The different species between the NC and DSS groups (**A**), between the VB8762 and DSS groups (**B**), between the DB8762 and DSS groups (**C**), and between the VB8762 and DB8762 groups (**D**). Wilcoxon rank-sum tests were used for statistical analysis.

Subsequently, to further investigate the potential gut microbiota composition difference between the NC, VB8762, DB 8762, and DSS groups, or between the VB8762 and DB8762 groups, the gut microbial landscape in all samples was assessed. The overall fecal microbiota comprised six main phyla, including Firmicutes, Bacteroidetes, Deferribacteres, Verrucomicrobia, Proteobacteria, and Actinobacteria. In terms of bacterial composition at the phylum level, all samples shared similar taxonomic communities and exhibited a relatively high abundance of the phyla Firmicutes and Bacteroidetes (Fig. 3D). No significant difference was found in the phylum-level gut microbiota composition between the NC, VB8762, DB 8762, and DSS groups, or between the VB8762 and DB8762 groups. At the species level, a total of 88 species were identified across all samples, and the top 12 species were shown in Fig. 3E. To confirm which species of bacterium was altered by VB8762 or DB8762 treatment, the differential species between the NC, VB8762, DB8762, and DSS groups were analyzed (Fig. 4). Specifically, 13, 14, and 11 differential species were identified between the NC, VB8762, DB8762, and DSS groups (Fig. 4A through C), respectively. Notably, there were some similar tendencies between VB8762 and DB8762 in the differential species, namely both the VB8762 and DB8762 groups had significantly more *Lactobacillus* sp. ASF360, *Mucispirillum schaedleri*, and *Subdoligranulum* unclassified but significantly fewer *Bacteroides thetaiotaomicron*, *Bacteroides massiliensis*, *Bacteroides caccae*, *Bacteroides uniformis*, and *Prevotella copri* compared with the DSS group. In addition, the VB8762 group had significantly more *Parabacteroides merdae*, *Bacteroides xylanisolvens*, *Odoribacter splanchnicus*, *Parabacteroides distasonis*, *Burkholderiales bacterium*, and *Aerococcus viridans* compared with the DSS group, while the DB8762 group had significantly more *Alistipes senegalensis* but fewer *Bacteroides fragilis* and *Bacteroides nordii* compared with the DSS group. Between the VB8762 and DB8762 groups, seven differential species were identified (Fig. 4D), displaying some differences in regulating some species of bacteria. Collectively, both VB8762 and DB8762 had significant effects on the gut microbiota composition in DSS-induced colitis rats.

### Both viable and dead *B. infantis* B8762 administration modulated rat fecal metabolome

**Fig 5:**
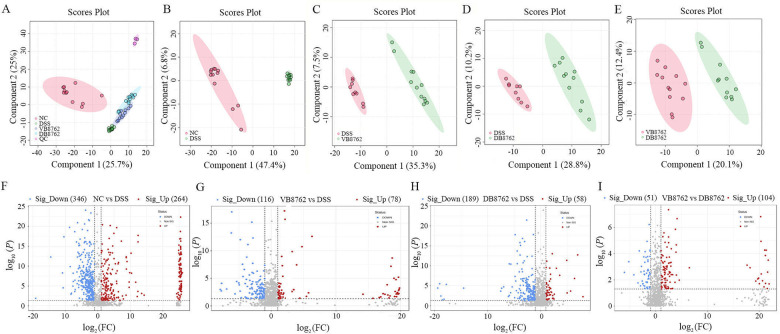
Comparison of the fecal metabolome of different pairwise comparisons. The partial least squares-discriminant score plots among all the four groups (**A**), between the NC and DSS groups (**B**), between the VB8762 and DSS groups (**C**), between the DB8762 and DSS groups (**D**), and between the VB8762 and DB8762 groups (**E**). The volcano plots between the NC and DSS groups (**F**), between the VB8762 and DSS groups (**G**), between the DB8762 and DSS groups (**H**), and between the VB8762 and DB8762 groups (**I**). Differential metabolites between groups were evaluated using analysis of variance at the 95% significance level (*P* < 0.05).

To further investigate the effects of VB8762 or DB8762 intervention on the fecal metabolome profile of DSS-induced IBD rat, inter-group differences in the fecal metabolome were analyzed. Partial least squares-discriminant analysis (PLS-DA), a supervised machine-learning algorithm that distinguishes the overall variability between samples, was performed to visualize changes in the fecal metabolome of every group. As expected, symbols representing groups of NC, DSS, VB8762, and DB8762 showed clear clustering trend, and the quality control samples also clustered separately as a group (Fig. 5A), indicating that the overall structure of the fecal metabolome varied between groups, and that VB8762 or DB8762 intervention indeed affected the fecal metabolome profile of DSS-induced IBD rat. Then, cluster analysis between groups (NC versus DSS; VB8762 versus DSS; DB8762 versus DSS; VB8762 versus DB8762) was performed by PLS-DA. The score plots of PLS-DA models are shown in Fig. 5B through E. Also, a clear cluster occurred in each pairwise comparison.

Furthermore, volcano plots between comparison groups are used to explore the differential metabolites (Fig. 5F through I). The cut-off thresholds for differential marker metabolites were fold change >2 and *P* < 0.05. A total of 610 (264 increased and 346 decreased, significantly), 194 (78 increased and 116 decreased, significantly), 247 (58 increased and 189 decreased, significantly), and 155 (104 increased and 51 decreased, significantly) differential metabolites were identified between groups (NC versus DSS; VB8762 versus DSS; DB8762 versus DSS; VB8762 versus DB8762), respectively.

**Fig 6:**
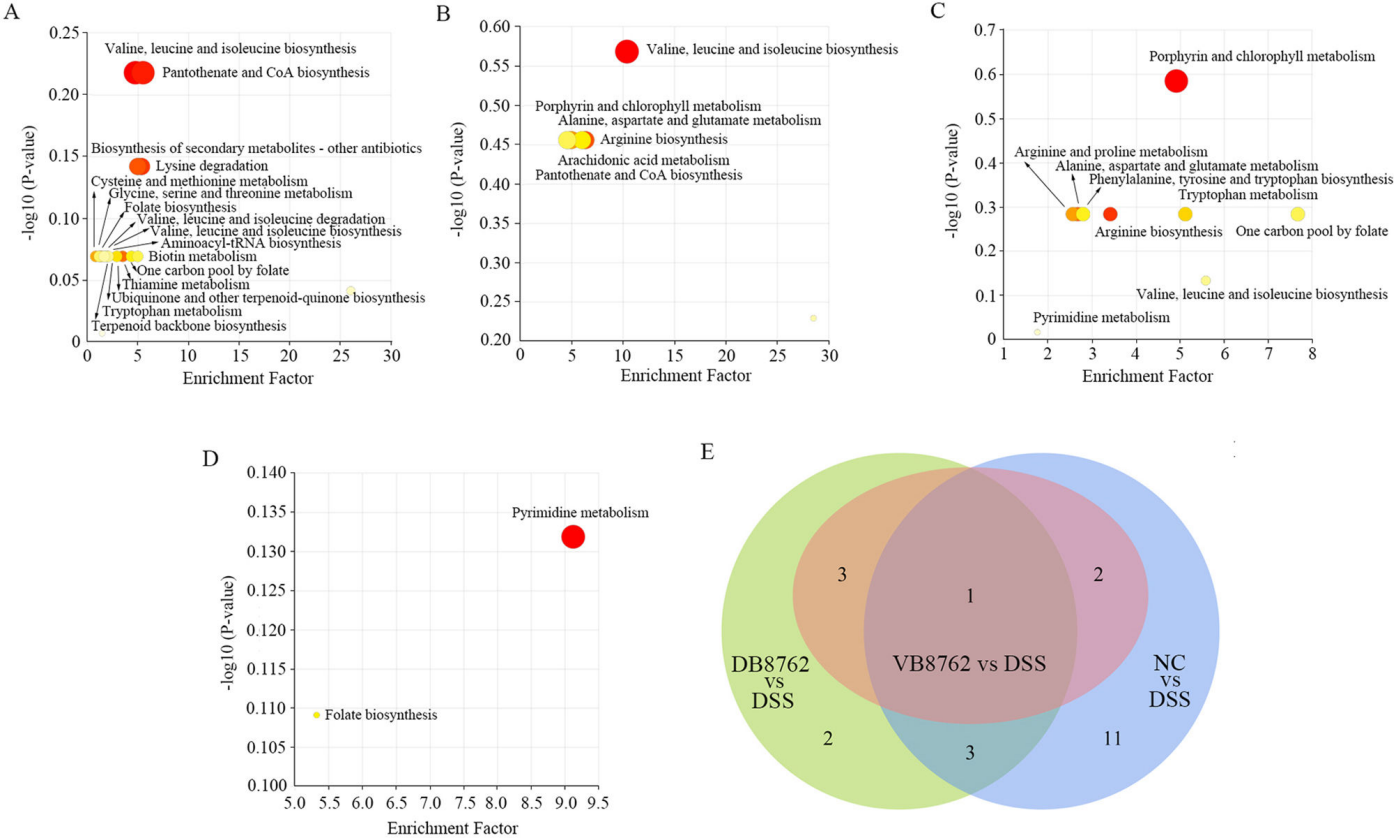
Significantly enriched metabolic pathways based on the differential metabolites between different pairwise comparisons. (**A**) NC versus the DSS group. (**B**) The VB8762 versus the DSS group. (**C**) DB8762 versus the DSS group. (**D**) VB8762 versus the DB8762 group. (**E**) Venn diagram of significantly enriched metabolic pathways of all the three pairwise comparisons of NC versus DSS, VB8762 versus DSS, and DB8762 versus DSS.

To further decipher the mechanisms of metabolic alterations of the samples, metabolic pathway enrichment analysis was performed based on the differential metabolites (Fig. 6A through D). In NC versus DSS comparison, 17 metabolic pathways were enriched, involving 27 different metabolites. In VB8762 versus DSS comparison, six metabolic pathways were enriched, involving nine different metabolites. In DB8762 versus DSS comparison, nine metabolic pathways were enriched, involving 16 different metabolites, and in VB8762 versus DB8762 comparison, two metabolic pathways were enriched, involving six different metabolites. Interestingly, the valine, leucine, and isoleucine biosynthesis pathway were enriched in all the three pairwise comparisons of NC versus DSS, VB8762 versus DSS, and DB8762 versus DSS (Fig. 6E), indicating that the pathway is an active pathway involved in the maintenance of intestinal barrier.

### Correlation between significantly differential gut microbiota and fecal metabolites

**Fig 7:**
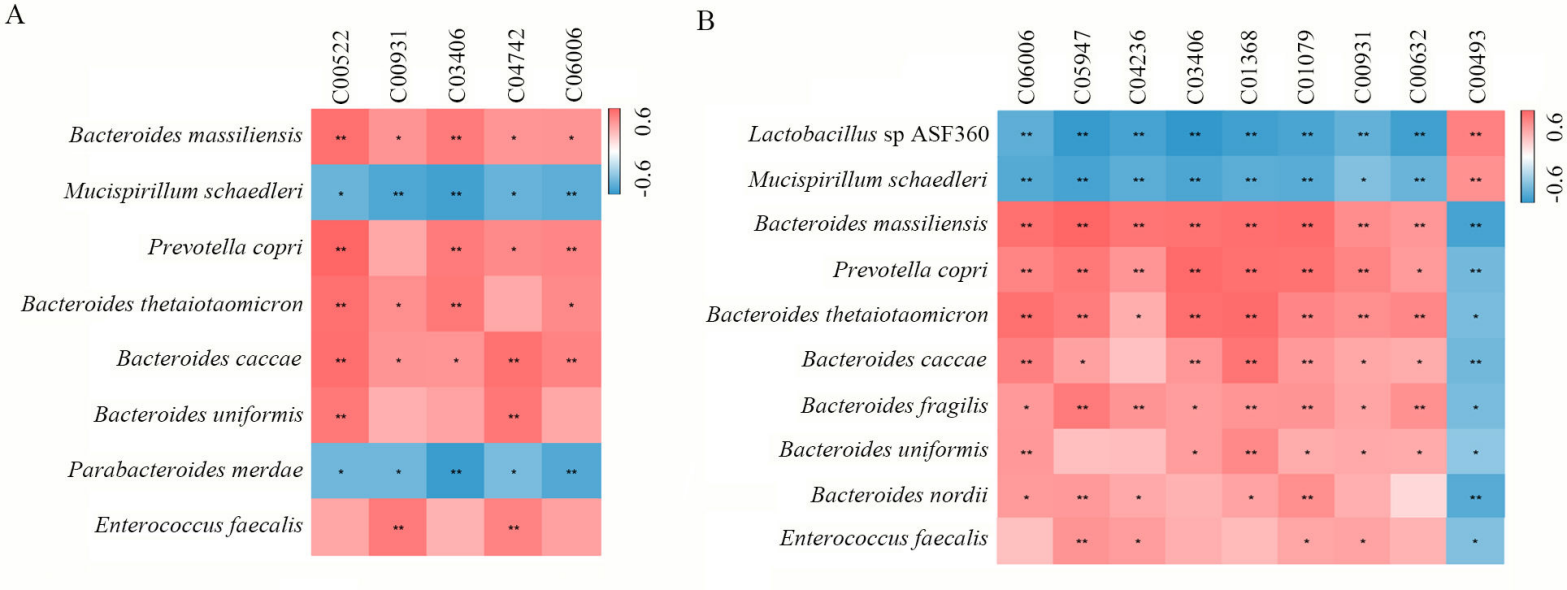
Correlation heat map of the Spearman’s rank correlation coefficient between gut microbiota and fecal metabolites. The color scale represents the strength of the correlation coefficient (ranging from 0.6 to −0.6). *P < 0.05; **P < 0.01.

A correlation analysis was performed to explore the relationship between rat gut microbiota and fecal metabolites. As shown in Fig. 7A, in the comparison of VB8762 vs DSS, both *Mucispirillum schaedleri* and *Parabacteroides merdae* showed significant negative correlation with all the five differential metabolites (C00522, C00931 C03406, C04742, and C06006), while *Bacteroides massiliensis*, *Prevotella copri*, *Bacteroides thetaiotaomicron*, *Bacteroides caccae*, *Bacteroides uniformis*, and *Enterococcus faecalis* all showed significant positive correlation with the five differential metabolites. In the comparison of DB8762 vs DSS (Fig. 7B), both *Lactobacillus* sp ASF360 and *M. schaedleri* showed significant negative correlation with C06006, C05947, C04236, C03406, C01368, C01079, C00931, and C00632, and significant positive correlation with C00493, while for *B. massiliensis*, *P. copri*, *B. thetaiotaomicron*, *B. caccae*, *Bacteroides fragilis*, *B. uniformis*, *Bacteroides nordii*, and *E. faecalis*, the situation was opposite.

## DISCUSSION

In the past years, numerous studies proved that many species of probiotics could ameliorate the symptoms and pathophysiology of colitis, and probiotic application is a good alternative to drugs for prevention and treatment of inflammatory bowel disease (39–46). Of these probiotics, *B. infantis* is one of the most commonly studied species. It was reported that *B. infantis* could exert protective effect in a mouse model of necrotizing enterocolitis (47). Javed et al. showed that *B. infantis* could alleviate 2,4,6- trinitrobenzenesulfonic acid (TNBS)-induced colitis (48). Supplementation with *B. infantis* could protect the goblet cells and epithelial cell layers. *B. infantis* BB-02 could alleviate the clinical symptoms of DSS-colitis, protect the colonic structure, and relieve edema (49). However, most studies focused on the effects of viable bacteria on IBD. Given the potential health risk with the use of live strains in certain groups, it is worth exploring whether the dead cells still exhibit the same beneficial function as the live strains. Therefore, in this study, as well as the viable *B. infantis* B8762, the effect of the heat-killed cells on IBD was also investigated in a DSS-induced IBD rat model. Surprisingly, our results found that both VB8762 and DB8762 administration exerted significant protective effect on DSS-induced IBD, as evidenced by a reduction in mortality, DAI score, body weight loss, as well as decreased histology score, which were accompanied by some changes in the rat gut microbiota, immune cytokines, and fecal metabolic pathways. Even so, there are some differences in modulating gut microbiota, immune response, and metabolic pathways between viable and dead bacterial cells. Collectively, the results support that both VB8762 and DB8762 are promising alternatives to drugs for the prevention and treatment of IBD.

Currently, the DSS model of colitis is a well-regarded and widely used rodent model of IBD due to its simplicity, reproducibility, and many similarities to human UC in terms of pathogenesis and therapeutic response (50). Similar to human IBD pathogenesis, gut microbiota dysbiosis associated with immune response is deeply involved in DSS-induced colitis. Therefore, DSS-induced rodent colitis is an ideal model for studying therapy strategies targeting colitis. Chronic, acute, and relapsing colitis models can be well established by adjusting the concentration of DSS and the frequency of administration. In our study, we successfully induced acute colitis in rats, resulting in symptoms of IBD including weight loss, rectal bleeding, diarrhea, and histological hallmarks by administering 3.0% DSS (wt/vol) through drinking water for 7 days. At day 7, the DAI scores of the DSS-induced groups were significantly higher than those of the NC group (Fig. 1C). After a 14-day period of bacteria intervention, a significant reduction in mortality, DAI score, body weight loss, and histology score was observed in both the VB8762 and DB8762 groups (Fig. 1B through F). In addition, the histopathological changes of rat colon tissues stained with H&E showed that both VB8762 and DB8762 could significantly alleviate DSS-induced colon damage, accompanied by a much reduced amount of inflammatory cell infiltration compared with the DSS group (Fig. 1F). Between the two groups of VB8762 and DB8762, although a rat in DB8762 group died, there were no significant differences in improving the indexes including DAI, body weight, and histology score. Together, these results indicated that both VB8762 and DB8762 administration could ameliorate the histopathological changes in the colon tissues of DSS-induced IBD rats.

Although the etiology of IBD is not yet fully known, it has been suggested that the onset and aggravation of the intestinal inflammation seem to be due to the dysregulation of the body’s immune responses with a parallel increase of pro-inflammatory cytokines, including IL-1β, IL-6, IL-8, TNF-α, and so on (51, 52). Of which, IL-1β was up-regulated in patients with UC, while IL-6 was up-regulated in patients with CD compared with healthy adults (53, 54). In addition, TNF-α induced massive apoptosis of colonic epithelial cells, thus increasing intestinal barrier cell permeability and aggravating intestinal inflammation (55). On the other hand, it was found that lack of IL-10, an anti-inflammatory cytokine, seemed to contribute to persistent intestinal inflammation (52). Therefore, in our study, three pro-inflammatory cytokines of IL-6, IL-1β, and TNF-α as surrogate markers of inflammation and IL-10 as a key anti-inflammatory cytokine were determined by enzyme linked immunosorbent assay (ELISA). Interestingly, compared with DSS group, there is a little difference between VB8762 and DB8762 in regulating these cytokines, that is, VB8762 downregulated all the three pro-inflammatory cytokines but DB8762 only downregulated IL-1β and TNF-α and did not alter the level of IL-6 (Fig. 2A through C). However, there is no significant difference between VB8762 and DB8762 in regulating all the three pro-inflammatory cytokines. Additionally, the anti-inflammatory cytokine IL-10 did not significantly change in all the four groups (Fig. 2D). Collectively, both VB8762 and DB8762 administration exerted an evident anti-inflammatory effect through downregulating anti-inflammatory cytokines, and VB8762 possessed a little stronger ability in regulating cytokines compared with DB8762.

Distinguished from common inflammatory diseases, the occurrence, aggravation, and remission of IBD are closely related to the gut microbiota. Numerous studies have proven that patients with IBD showed reduced biodiversity in gut microbial structure and composition called dysbiosis, characterized by the reduction of beneficial commensal microflora and the increase of pathogenic bacteria (56, 57). Here, we used whole-metagenome shotgun sequencing to investigate the potential changes in gut microbial diversity and composition after bacterial administration. Alpha-diversity measurement results, based on Shannon and Simpson indexes, showed that both VB8762- and DB8762-treated rats exhibited a fecal microbiota with significantly higher diversity than that of the DSS-treated rats (Fig. 3A and B). As for beta-diversity, the rats of both VB8762 and DB8762 groups harbored significant difference in the fecal microbiota structure from DSS group rats through PCoA, indicating that both VB8762 and DB8762 administration markedly transformed the biological community structures disrupted by DSS (Fig. 3C). Moreover, it was observed that VB8762 application showed a little stronger ability in modulating the gut microbiota composition than DB8762.

The modulating capacities of VB8762 and DB8762 were further evaluated at different levels of phylum and species, respectively. At phylum level, both VB8762 and DB8762 interventions did not alter the gut microbiota composition, which is similar to many previous reports that the gut microbiota at phylum level has not shown obvious changes subjected to probiotic administration (44, 58–60). At species level, there were some similar tendencies between VB8762 and DB8762 in the differential species (Fig. 3E). A relatively higher abundance of *B. massiliensis* and *B. thetaiotaomicron* and a relatively lower abundance of *Lactobacillus* sp. ASF360, *M. schaedleri*, and *Subdoligranulum* unclassified were observed in the DSS group compared with the VB8762 or DB8762 group (Fig. 3E and 4B and C). Notably, *B. massiliensis* has been shown to be prevalent in colitis (61). *Lactobacillus* is generally considered a beneficial genus in the gut that plays health functions. Many species of *Lactobacillus* produce a large number of metabolites to regulate intestinal homeostasis and maintain normal intestinal barrier functions (62–64). *M. schaedleri,* a spiral-shaped bacterium colonizing the mucus layer of the gastrointestinal tract of rodents, restricted *Salmonella* infection and inhibited virulence factor expression, then conferring protection against *Salmonella* colitis in mice (65). *Subdoligranulum*, which belongs to the *Clostridium* cluster of the phylum Firmicutes, is one of the most important butyrate producers in intestinal tract. Gut-microbial butyrate is a short-chain fatty acid (SCFA) of significant physiological importance than the other major SCFAs (acetate and propionate) (66). It has been revealed that *Subdoligranulum* is beneficial for necrotizing enterocolitis by influencing the bacterial phages and butyrate production (67). In conclusion, at species level, more differential species were observed between VB8762 and DSS than those between VB8762 and DSS, indicating VB8762 intervention also showed stronger ability in modulating the gut microbiota at species level than DB8762.

Changes in the gut microbiota composition would naturally result in alterations in the fecal metabolome and related metabolic pathways (68, 69). The good cluster results indicated that VB8762 or DB8762 intervention indeed altered the fecal metabolome profile of DSS-induced IBD rat (Fig. 5C and D). But the clear cluster between the VB8762 and DB8762 groups reflected the difference in the fecal metabolic profile (Fig. 5E). More differential metabolites and differential metabolic pathways were identified in the DB8762 group compared with the VB8762 group (Fig. 5G, H, 6B, C, and E), indicating that DB8762 intervention had a stronger ability to induce more changes in the metabolites production than VB8762, which was inconsistent with the lower regulating capability of gut microbiota composition. The discrepant results might be due to the difference in the production of metabolites of some differential species of bacteria between the VB8762 and DB8762 groups. However, there were four metabolite pathways of overlap between the two pairwise comparisons of VB8762 vs DSS and DB8762 vs DSS, suggesting some similarities in changing fecal metabolome between VB8762 and DB8762 intervention.

Additionally, the present study found a significant and valuable correlation between differential gut microbiota and fecal metabolites. Between VB8762 vs DSS and DB8762 vs DSS, the present study showed a similar correlation heap map profile. In the comparison of VB8762 vs DSS (Fig. 7A), two potential probiotic bacteria, i.e., *M. schaedleri* and *P. merdae*, negatively correlated with all the five differential metabolites. In the comparison of DB8762 vs DSS (Fig. 7B), two potential probiotic bacteria, i.e. *Lactobacillus* sp. ASF360 and *M. schaedleri*, negatively correlated with eight differential metabolites but positively correlated with one metabolite of C00493, which is associated with phenylalanine, tyrosine, and tryptophan biosynthesis. The results indicated that both viable *B. infantis* B8762 and dead cell administration can bring a potential effect on regulating gut bacterial metabolism.

In 2021, the International Scientific Association for Probiotics and Prebiotics defined postbiotics as “preparation of inanimate microorganisms and/or their components that confers a health benefit on the host” (70). Overall, our data showed that the heat-killed *B. infantis* B8762 conferred a certain extent of IBD-associated symptom alleviation effect, which could be comparable to that offered by VB8762. Hereby, the heat-killed *B. infantis* B8762 can be confirmed to be a postbiotic. As previously reported (71), postbiotics have several advantages over probiotics, e.g., potential safety issues of spreading of the intrinsic antibiotic resistance genes and other virulence genes existing in live probiotics to the gut microbes of host or the possibility of gaining access to extraintestinal organs of certain groups like neonates and vulnerable populations and causing life-threatening conditions like septicemia. Our results supported that the postbiotic DB8762 could be a promising alternative to probiotics to be applied in the prevention and treatment of IBDs due to its minimal safety concerns. However, more in-depth and precise experiments need to be performed to explore the exact mechanism of action of the viable *B. infantis* B8762 and heat-killed cells in relieving colitis.

### Conclusions

In this study, it was proven that both viable *B. infantis* B8762 and heat-killed cells could alleviate DSS-induced colitis in rats by reducing colonic mucosal damage, modulating immune responses, restoring gut microbiota diversity, and inducing some fecal metabolic responses. The findings of our study support that the heat-killed *B. infantis* B8762, as a postbiotic, could be a promising alternative to probiotics to be applied in the prevention and treatment of IBDs. However, the specific mechanism of action requires further study.

## MATERIALS AND METHODS

### Animals

Male Wistar rats (6 weeks of age, weighing 175–250 g), specific pathogen-free (SPF), were purchased from Charles River Labs Co., Ltd (Beijing, China) and group-housed in an individually ventilated cage (IVC) system (Shandong SHINVA Medical Device Co. Ltd, Shandong, China). Throughout the acclimatization and study periods, all animals were maintained on a 12-h light-dark cycle (lights on at 8:00 and lights off at 20:00) with an ambient temperature of 21°C ± 2°C and a relatively constant humidity of 45 ± 10% under SPF conditions. The rats were fed sterile chow diets and provided tap water *ad libitum*. The body weight of each rat, along with its food consumption and water intake, was recorded daily as a general day-to-day indication of their health and well-being.

### Bacterial strains and reagents

*B. infantis* B8762 was obtained from the the Lactic Acid Bacteria Collection Center of the Inner Mongolia Agricultural University. *B. infantis* B8762 was activated in M17 (Qingdao HopeBiol Co., Qingdao, China) liquid media at 37°C for 24 h anaerobically. After culture, the cells were collected by centrifugation at 4,000 *g* for 15 min at 4°C and resuspended in sterile phosphate buffer saline buffer (PBS, 0.8% NaCl, 0.02% KH_2_PO_4_, 0.115% Na_2_HPO_4_, 1% tryptone, and 0.1% sodium glutamate, pH 7.0). Subsequently, half of the above bacterial suspension was transferred to another container and inactivated at 121°C for 15 min. The viable and inactivated bacterial suspensions were prepared freshly and adjusted to the concentrations of 4 × 10^9^ colony-forming units (CFU) per milliliter before every use. DSS with molecular weight of 36–50 KD was purchased from MP Biomedicals (Santa Ana, CA, USA), and all the other chemicals used in study were obtained domestically and analytically pure.

### DSS-induced colitis

Once bought back, the rats were firstly acclimated for 7 days in IVC, followed by the treatment with DSS. Rats were grouped into NC and three DSS-treated groups according to random principle. NC group received only normal tap water throughout the study. The acute DSS-colitis model was induced as previously described (72). Specifically, the rats was administered 3% (wt/vol) DSS in their drinking water *ad libitum* for 7 days. Following cessation of DSS induction, the DSS-colitis rats were divided into three groups as follows (*n* = 12 per group, three rats per cage): viable *B. infantis* B8762 (VB8762) group receiving a 2-week viable VB8762 (4 × 10^9^ CFU per day for each rat dissolved in 2 mL saline) intervention via intragastric gavage; dead *B. infantis* B8762 (DB8762) group receiving the same intervention as VB8762 group except using dead bacteria instead of viable ones; and DSS group receiving 2 weeks of equal volume of saline administration.

During the intervention, rats were checked daily for morbidity, and the DAI was used to assess induced colitis severity for every rat according to three pathological features (weight loss, stool consistency, and hematochezia) as described previously with minor modifications (73), each scored on a scale from 0 to 4 according to a scoring standard (Table S1). Scores for all three pathological features were summed to give DAI scores per rat. At the time point of 21 days, all the rats were euthanized and stool, blood, and colons were sampled.

### Histological analysis

At the time point of 21 days, fresh feces of every rat were collected and stored at −80°C, and all the rats were anesthetized with sevoflurane and killed for blood and colon collection. The whole colon was pulled out carefully, detached from the surrounding mesentery. Then, distal colon samples (1–2 cm) were transected and dissected, emptied of their contents, washed with sterile PBS and fixed in 10% buffered formaldehyde. Slices were sectioned using a Leica RM2235 system (Leica, Wetzlar, Germany), dehydrated by gradient ethanol and xylene, embedded in paraffin, stained with H&E, and visualized using video camera microscope (Leica, Wetzlar, Germany), as described previously (39). Histological scoring was performed according to a previous study to evaluate DSS damage of the colon by an unbiased observer in a blinded manner (40, 72). Briefly, the histological damage scores were summed by four histological parameters including goblet cell depletion, hyperplasia of connective tissue, degree of inflammatory cell infiltration, and edema of cells, with each parameter assigned one to four grade levels (0–4).

### Measurement of cytokines

At the time point of 21 days, blood samples were collected from abdominal aorta when euthanized. One to 2 mL blood samples were collected into a coagulation tube and allowed to coagulate for 1–2 h at room temperature. Following low-speed centrifugation at 2,000 rpm for 5 min, the upper serum layer was taken and stored at −80°C for further ELISA assay. The concentrations of the cytokines of IL-6, IL-1β, IL-10, and TNF-α were quantified using ELISA kits (Catalog number 210305, Signalway Antibody, College Park, Maryland, USA) according to the manufacturer’s instructions. Briefly, 100 µL of serum samples was incubated for 2 h at 37°C to be combined with the antibodies coated in the plate in advance, followed by incubation with detection antibodies (1:100) for 1 h. After that, streptavidin conjugated with horseradish peroxidase was added and incubated for 1 h. Subsequently, 90 µL of substrate solution was added for color reaction. Finally, reaction was stopped by adding 50 µL H_2_SO_4_ and detected using a microplate reader at 450 nm. The concentrations were calculated according to the standard curves.

### Analysis of fecal microbiota by metagenomic sequencing

Total fecal genomic DNA was extracted from feces of every rat at the time point of 21 days using the QIAamp Fast DNA Fecal Mini Kit (Qiagen GmbH, Hilden, Germany) according to the manufacturer’s instructions, and DNA purification was performed. Then, the concentration and integrity of DNA were measured by NanoDrop One spectrophotometer (Thermo, USA) and agarose gel electrophoresis. Sequencing libraries were constructed using the NEBNext Ultra DNA Library Prep Kit (New England Biolabs, Inc., Ipswich, MA, USA). Complete paired-end metagenome sequencing was performed on the Illumina NovaSeq PE150 platform at the Beijing Novogene Bioinformatics Technology Co., Ltd., China. After removing low-quality and potentially contaminating sequences, HUMAnN2 was used for metagenomic analysis.

Raw sequence data generated in this study are deposited at NCBI-SRA (BioProject: PRJNA1070846; https://www.ncbi.nlm.nih.gov/bioproject/PRJNA1070846, accessed on 31 December 2026).

### Untargeted metabolomics analysis by UHPLC-Q-TOF MS

In preparing samples for analyzing non-volatile component profiles, for each feces sample, 1 mL of precooled methanol:water solution (4:1, vol/vol) was added to 0.2 g of naturally thawed feces, vortexed for 30 s. The mixtures were sonicated for 30 min on ice water, followed by centrifugation at 4,000 *g* for 10 min at 4°C, then the supernatant was pipetted to another 2 mL tube. The precipitation was extracted again with 0.5 mL methanol:water solution as above, then the two supernatants were mixed and centrifuged at 10,000 *g* for 5 min at 4°C. The supernatant was collected and filtered through 0.22 µm water insoluble microporous membrane filter before analysis by ultra-high performance liquid chromatography coupled with electrospray ionization/quadrupole-time-of-flight mass spectrometry (UPLC-ESI-Q-TOF-MS) and mainly used for analyzing the metabolites. Meanwhile, quality control sample was prepared by pipetting certain amount of every extraction samples and mixing thoroughly.

The pre-treated feces samples were loaded onto an Acquity UPLC system coupled to a Waters Q-TOF mass spectrometer (Water Corp., Milford, MA, USA) for analysis. An Acquity UPLC HSS T3 column (2.1 mm × 100 mm ×1.7 µm, Waters Corp.) was used for separation with a flow rate of 0.3 mL/min. The column temperature was maintained at 40°C throughout the analysis. A 4 μL of each sample was injected into the system and triplicate repeats were performed.

An ESI source that was operated in both positive and negative ionization modes (ESI+ and ESI−) was used to generate ions for analysis. The mass spectrometry conditions were set according to a previous study (41). Under positive ion mode, the mobile phase consisted of 0.1% formic acid in ultrapure water (A1) and 0.1% formic acid in acetonitrile (B1), and under negative ion mode, the mobile phase consisted of 0.1% ammonium hydroxide in ultrapure water (A2) and acetonitrile (B2). A gradient elution program was applied as follows: 0–2.5 min, 95%–84% A; 2.5–12 min, 84%–45% A; 12–15 min, 45%–35% A; 15–18 min, 35%–28%; 18–20 min, 28%–15%; 20–25 min, 15%–2%; 25–28 min, 2%–95%. Leucine-enkephalin was used as the lock mass for positive ion mode (556.2771［M + H］^+^) and negative ion mode (554.2615［M − H］^-^) to ensure the reproducibility and accuracy of acquired data. Raw data obtained by UPLC-Q-TOF-MS were collected in continuum mode and processed using the Progenesis QI software (Waters Corp.). Several data pre-processing procedures including peak alignment, peak identification, and deconvolution were performed. Mass accuracy was set to 10.0 ppm and *P*-value cut-off was set to 0.05 in mummichog implementation. Statistical analysis module was applied to do data filtering and preprocessing. Metabolite abundances were filtered by mean intensity and preprocessed by sum normalization and range scaling. Data from positive mode and negative mode were mixed and processed together.

### Statistical analysis

The results of body weight, immune factors, and histological and DAI scores were analyzed using GraphPad Prism 8.0 (GraphPad Software, San Diego, CA, USA). Student’s *t*-test (unpaired, two-tailed) or Wilcoxon rank-sum test was used to determine levels of significance for comparisons between two groups. The Shannon index, Simpson index, PcoA, and PLS-DA were performed with R packages (vegan, optparse, and ggpubr). Volcano plots were used to identify differential metabolites, and significantly differential metabolomic features were selected according to: fold change ˃2, variable influence on projection score ˃1, and *P* < 0.05. The Venn diagram was plotted using the OmicStudio tools at https://www.omicstudio.cn. The metabolic pathway enrichment analysis was performed using the pathway module in Metaboanalyst 5.0 (https://www.Metaboanalyst.ca). Correlation analyses between significantly differential gut microbiota and fecal metabolites were performed using Spearman’s rank correlation coefficient. Data are presented as mean ± SD. Statistical significance is indicated as follows: *P < 0.05; **P < 0.01; ***P < 0.001; ****P < 0.0001 and NS means no significance.

## Data Availability

The data set supporting the conclusions of this article is available from the corresponding authors on reasonable request.

## SUPPLEMENTAL MATERIAL

Supplemental material spectrum.03509-23-s0001.docx Table S1: Scoring standard for Disease Activity Index.

ASM does not own the copyrights to Supplemental Material that may be linked to, or accessed through, an article. The authors have granted ASM a non-exclusive, world-wide license to publish the Supplemental Material files. Please contact the corresponding author directly for reuse.
