# Supplementary material for: Both viable Bifidobacterium longum subsp. infantis B8762 and heat-killed cells alleviate the intestinal inflammation of DSS-induced IBD rats
Source: Microbiol Spectr. 2024 Apr 22;12(6):e03509-23. doi: 10.1128/spectrum.03509-23 (PMC11237488; doi:10.1128/spectrum.03509-23)
Supplement: Supplemental material — Table S1: Scoring standard for Disease Activity Index. [file spectrum.03509-23-s0001.docx]

**Supplementary Table S1**

Scoring standard for Disease Activity Index (DAI)

| Score | Weight loss | Stool consistency | Blood stool |
| --- | --- | --- | --- |
| 0 | no loss | normal | no blood |
| 1 | 1-5% | loose stool |  |
| 2 | 5-10% | watery diarrhea | presence of blood |
| 3 | 10-20% | slimy diarrhea, little blood |  |
| 4 | >20% | severe watery diarrhea with blood | gross bleeding |
